# Factors associated with prolonged viral shedding in COVID-19 infection: A retrospective cohort from Karachi, Pakistan

**DOI:** 10.1371/journal.pone.0336774

**Published:** 2025-11-13

**Authors:** Lokesh Kumar, Ishfaque Ahmed, Chanchal Kumari, Nosheen Nasir

**Affiliations:** 1 Department of Medicine, Section of Internal Medicine, Aga Khan University Hospital, Karachi, Pakistan; 2 Department of Medicine, Section of Infectious Diseases, Aga Khan University Hospital, Karachi, Pakistan; 3 Department of Medicine, Jinnah Postgraduate Medical College, Karachi, Pakistan; Children's National Hospital, George Washington University, UNITED STATES OF AMERICA

## Abstract

**Background:**

The implications of prolonged viral shedding in COVID-19 are of major public health concern. There are several studies elucidating the impact on transmission; there is a lack of data on outcomes. The objective of this study was to identify factors associated with prolonged viral shedding and its impact on disease outcomes in COVID-19.

**Methods:**

This retrospective cohort was conducted on hospitalized throat swab-PCR confirmed COVID-19 patients admitted between March 01, 2020, and June 07, 2020, at the Aga Khan University Hospital in Karachi, Pakistan. Demographic, treatment and successive SARS CoV-2 PCR data were extracted from medical records using a structured proforma. Prolonged viral shedding was defined as PCR positivity greater than or equal to 15 days from the first positive PCR. Outcomes studied included in-hospital mortality, length of stay, and requirement of mechanical ventilation.

**Results:**

Out of 435 patients, only 110 could be assessed for time to negativity. 47 patients (42.7%) had viral shedding for more than 15 days compared to 63 (57.3%) patients with viral shedding for less than 15 days. The median duration of time to negativity in the prolonged shedding group was 25 days compared to 9 days in the other group. The median age was 54, and it was similar in both groups. Most of the patients had mild diseases in both groups. There was no statistically significant difference between either of the groups in terms of in-hospital mortality (2/47 versus 1/63) and length of stay (9 versus 8) days.

**Conclusion:**

This study did not find any factors associated with prolonged viral shedding in COVID-19, and there was no impact of prolonged viral shedding on in-hospital mortality.

## Introduction

The outbreak of COVID-19 caused by SARS-CoV2, was first reported in Wuhan, China, near the end of the year 2019 [1,2]. The World Health Organization (WHO) declared it pandemic in February 2020 [3]. Since then, there have been more than 252,000,000 cases with more than 5 million deaths reported globally [4]. Epidemic prevention and control have been the greatest challenge and one of the reasons is that the duration of the SARS-CoV2 viral shedding from infected persons is uncertain [5]. While RT-PCR is used for establishing the diagnosis of COVID-19, persistent viral shedding has been reported and a positive RT-PCR does not always mean infectivity [6]. Though SARS-CoV2 has less mortality than MERS-CoV, it is far more infective and transmissible. Most patients’ PCR has been reported negative within three weeks of the infection, but viral shedding has been documented for more than 2 months after the infection in some patients [7–10]. Genomic studies showed that during the first wave lasting from April 2020 to June 2020, frequent COVID isolated were B.1 followed by B.1.1.1, B.1.36 and A. The common clades isolated were GH and GR, that are known to cause severe disease [11,12]. Viral Shedding time (VST) has been variably reported from different parts of the world. A retrospective study conducted in Beijing, China defined viral persistence as PCR positivity after 11 days of the first positive PCR, while another by Xu et al. described prolonged viral shedding as a positive PCR after 15 days of the initial positivity [7,13]. Several other studies have reported SARS Cov-2PCR positivity for as long as 60 days [7,9,13–15]. In one study, viral shedding was positive for 83 days in the upper respiratory tract [16]. The factors that contribute to prolonged viral shedding have remained a matter of debate. A study done at Zhejiang University, China, identified male sex, advancing age, hypertension, severe illness, steroids, and invasive ventilation as the factors associated with prolonged viral shedding [7]. *Yan et al.* also described age as an independent factor associated with persistence of the virus in the respiratory specimen [17]. Whether this prolonged positivity should be considered as clinically significant shedding remains a question. A meta-analysis showed that there was no live virus in the specimen beyond 9 days [10]. Although studies based on virus culture have concluded that the viral shedding from recovered immunocompetent patients does not have infectious potential; its implications on transmission and duration of infectiousness of immunocompromised patients needs to be determined.

Among the many challenges that come across due to prolonged virus shedding are the decision to discontinue isolation and whether to continue antiviral treatment. This has impact on resources in low-middle income countries (LMICs) as there are problems in getting repeat Polymerase Chain Reaction (PCR) testing in affected persons due to the cost incurred and similarly continuation of home isolation has direct and indirect impact on economic well-being of people in these countries. However, there is lack of data on viral shedding from LMICs to define the adequate time interval for testing in such patients if required and whether it has any significant bearing on disease outcomes Hence, we decided to conduct this study on various factors that may be related to prolonging the viral shedding and their significance on the outcome of the patients.

## Methods

A prospective cohort study was conducted on all adult patients of age greater than or equal to 18 years who had throat swab tested positive for SARS Cov-2 and were admitted at the Aga Khan University Hospital, Karachi, The study was conducted after approval from the Aga Khan University Ethical Review Committee (ERC number: 2020-4877-11759) and a verbal consent from the patients. Data including age, gender, co-morbid conditions, treatment, and classification of disease severity were collected from electronic medical records on a pre-designed proforma from 01/10/2020 to 25/12/2020. All the data was accessible by the primary author and kept on his personal computer to maintain patient’s confidentiality. All adult patients with a positive SARS CoV-2 PCR, and those who follow up on negative PCR were documented, were included in the study. Patients with no negative PCR, due to financial issues or changes in national guidelines, were excluded.

We followed all hospitalized patients who tested positive for COVID 19 by PCR from 1st March 2020–7th June 2020. Patients were followed until they had two consecutive RT-PCR for SARS-Cov-2 reported negative. The duration of viral RNA shedding was defined as the number of days from symptom onset to persistent negative detection of respiratory tract specimens. Prolonged viral shedding was defined as PCR positivity on 15 days or greater after the date of the first positive PCR [7]. All statistical analyses were performed using SPSS (Statistical Package for the Social Sciences) version 21.0 software (SPSS Inc). A p-value of <0.05 was considered statistically significant. All categorical variables like gender, co-morbid, type of treatment, and clinical outcome were reported as proportions or percentages. Continuous variables like age, hospital stay, ICU stay were reported as mean, median, and interquartile ranges as appropriate. The Chi-square test or Fisher exact test was used to determine the association between two categorical variables like disease severity or type of treatment with prolonged viral shedding.

## Results

A total of 435 COVID-19 patients were admitted from March 2020 to June 2020. 310 patients were excluded from the study as repeat PCR was not performed because of the revision on the national guidelines, which instituted discontinuation of repeat testing in immunocompetent patients. 15 patients were excluded as the data was not complete for them, so 110 patients’ data was included in the final analysis.

In those patients who had viral shedding of more than 15 days, the median age was 54 years (IQR 34–62), and of those 65.9% (n = 31) were males, while in the other group, the median age was 49 years (IQR 39–62) and gender predominance was similar (males 63.4%) (Table 1). The median length of hospital stay was nine days in the first group with prolonged shedding (IQR 3–14 days) and in the patients who did not have prolonged shedding, the median length of hospital stay was eight days (see Tables 1 and 3). Most of the patients had mild disease with prolonged viral shedding (42.5% n = 20), meanwhile the majority of patients in the comparison group had the moderate disease (39.6% n = 25) (Table 1) but this was not seen to be statistically significant (p-value 0.190). We found a similar frequency with reference to the co-morbid conditions in both groups. Most frequent co-morbid conditions were hypertension (17 cases 36.1% vs 25 cases 39.6%) and diabetes (13 cases 27.6% vs 20 cases 31.7%) (Table 2) [18].

**Table 1:** Baseline Characteristics of patients with viral shedding>= 15 days compared with those with viral shedding less than 15 days.

Variables	Viral Shedding >= 15 days	Viral Shedding < 15 days	p-value
Median Age (IQR)	54 (34-62)	49 (39-62)	0.973
**Gender**			0.789
Male	31 (65.9%)	40 (63.4%)	
Female	16 (34.1%)	23 (36.6)	
**Severity of Illness** **(old classification)**			
Mild	*20 (42.5%)*	22 (34.9%)	0.415
Moderate	13 (27.6%)	25 (39.6%)	0.190
Severe	10 (21.2%)	13 (20.6%)	0.935
Critical	5 (10.6%)	3 (4.7%)	0.283
**(new classification)**			
Non-Severe Severe	32 (68%) 15 (31.9%)	47 (74.6%) 16 (25.4%)	0.452

**Table 2:** Comparison of Co-Morbidities of patients with viral shedding>= 15 days compared with those with viral shedding less than 15 days.

Comorbidity	Viral Shedding >= 15 days	Viral Shedding <15 days	p-value
Diabetes Mellitus	13 (27.6%)	20 (31.7%)	0.644
Hypertension	17 (36.1%)	25 (39.6%)	0.708
Chronic Kidney Disease	3 (6.3%)	4 (6.3%)	
Chronic Lung Disease	7 (14.8%)	3 (4.7%)	0.095
Immunocompromised	1 (2.1%)	1 (1.5%)	
Auto-Immune Disease	2 (4.2%)	1 (1.5%)	
Chronic Liver Disease	2 (4.2%)	1 (1.5%)	
Coronary Artery Disease	7 (14.8%)	11 (17.4%)	
Malignancy	3 (6.3%)	5 (7.9%)	
Cerebrovascular Accident	1 (2.1%)	4 (6.3%)	

**Table 3:** Comparison of Treatment and outcomes of patients with viral shedding>= 15 days compared with those with viral shedding less than 15 days.

Variables	Viral Shedding >15 days	Viral Shedding <15 days	p-value
Invasive Ventilation	7 (14.8%)	6 (9.5%)	0.338
Non-invasive mechanical Ventilation	10 (21.2%)	14 (22.2%)	0.905
Oxygen Supplementation	20 (42.4%)	29 (46%)	0.717
Systemic Steroids	23 (48.9%)	25 (39.6%)	0.333
Hydroxychloroquine	12 (25.5%)	22 (34.9%)	0.292
Antivirals	3 (6.3%)	1 (1.5%)	0.184
Tocilizumab	6 (12.7%)	10 (15.8%)	0.648
Plasma therapy	0	1 (1.5%)	
Intravenous Immunoglobulin	0	1 (1.5%)	
Chloroquine	3 (6.3%)	3 (4.7)	
**Outcomes**			0.392
Alive	44 (93.6%)	62 (98.4%)	
Dead	2 (4.2%)	1 (1.5%)	
Median LOS (IQR)	9 (3-14)	8 (9-11)	

In the group of patients with persistent viral shedding, 23 (48.9%) patients received steroids, and 20 (42.4%) patients required supplemental oxygen. On the contrary, the patients with viral clearance of less than 15 days, 25 (39%) patients received steroids, and 29 (46%) patients were given supplemental oxygen support. 6 (12.7%) patients received treatment with tocilizumab, and 3 patients (6.3%) received treatment with remdesivir in the first group while in the second group the number was 10 (15.8%) and 1 (1.5%) patient, respectively. None of the patients in the first group required plasma therapy or IVIG, while in the second group only one patient (1.5%) received plasma, and one received IVIG (Table 3). 12 patients (25.5%) with prolonged viral shedding commenced on HCQ and 22 patients (34.9%) with normal viral shedding received it. 93.6% (n = 44) recovered from illness and 4.2% (n = 2) patients died in the first group and 98.4% (n = 62) of patients were sent home alive and only 1 death was documented in the comparison group (Table 3).

## Discussion

Our study did not find any factor associated with prolonged viral shedding, be it co-morbid illnesses or the treatment that the patients received for COVID-19. More importantly, our study did not show an association between prolonged viral shedding with mortality. During literature review, no such study was found on whether viral shedding has any interrelation with mortality or not.

Xu et al conducted a retrospective study on the factors that contribute to viral shedding. They evaluated 113 patients from 2 different hospitals in China and found that several risk factors were associated with prolonged viral shedding. They concluded that 74.3% of patients could clear the virus with a median duration of 15 days. They compared the two groups, i.e., with viral shedding of 15 days and more than 15 days, based on epidemiology, treatment and outcome. Their study concluded that prolonged viral shedding was associated with male gender (p = 0.009), old age (p = 0.033), hypertension as co-morbid (p = 0.009). In addition to this, the patients who had severe disease on presentation had delayed clearance (p = 0.049) [7]. Regarding treatment, corticosteroid and invasive mechanical ventilation were strongly associated with delayed viral clearance (p = 0.025 and 0.006, respectively) [7]. A study by *Li et al.* supports corticosteroids as a risk factor for delayed viral clearance [13]. On the contrary, another cohort conducted in China failed to demonstrate any association of corticosteroid, concomitant hypertension, male gender or disease severity as causative factors for delayed viral shedding. In contrast, old age and lack of ritonavir/lopinavir treatment were associated with prolonged clearance [16]. In our study, we did not find any association of gender, co-morbidities, and disease severity with prolonged viral shedding and that is in accordance with a few published literatures [16,19,20]. Similar results were obtained from the cohort published in the European Respiratory Journal [17]. A retrospective cohort published in the International Journal of Infectious diseases showed that the median duration of viral shedding was 17 days. Furthermore, the results concluded that gender (p-0.123), two common co-morbidities, i.e., hypertension (p = 0.573) and diabetes (p = 1.000), and disease severity (p = 0.296), had no impact on viral shedding [19]. The study conducted by Cao *et al.* reported similar findings as our study with regards to gender (p = 0.33) and severity of illness (p = 0.30) which were found to have no role in prolonging the RNA shedding of SARS-CoV-2. Our study did not demonstrate interdependence of any sort of treatment, including supplemental oxygen, corticosteroids, antivirals and invasive or non-invasive mechanical ventilation with persistent viral shedding of more than 15 days.

The outcomes we measured in our study were the length of hospital stay and mortality. There was no significant difference in results in both groups (p = 0.392). On the review of the literature, no such study was found in which the relationship between prolonged viral shedding and its impact on length of hospital stay and mortality was studied.

## Conclusion

To conclude, our study did not find any factor that prolongs viral shedding in the patients with SARS-CoV-2 infection, be it age, gender, co-morbidity or the disease severity. This cohort also showed that viral clearance is not dependent on any sort of in-hospital treatment, from simple supplemental oxygen therapy to remdesivir and tocilizumab. Furthermore, our study suggested that there is no impact of viral shedding either in the length of hospital stay or the mortality in the hospital.

### Limitations

Our study has a couple of limitations. The major limitations of our study were; it’s a single-center study with a limited sample size. Because of that, one-way and multifactor regression analysis could not be performed as it may lead to unreliable estimates. Due to revision in the national guidelines at the time of data collection, we had to exclude more than half of the sample size. Apart from that, due to unavailability of multiplex respiratory panels, we could not exclude co-infection with other common respiratory infections and their impact on the study results.
